# Compound Fracture of the Inferior Maxilla Treated by Wire Suture

**Published:** 1893-03

**Authors:** T S. Carter

**Affiliations:** Dental Surgeon to the Leeds General Infirmary


### SELECTIONS.

### Compound Fracture of the Inferior Maxilla Treated by Wire Suture.

BY T S. CARTER, L.D.S R.C.S., ENG.
Dental Surgeon to the Leeds General Infirmary.

The obstacles which have to be overcome in order to maintain fractured portions of the inferior maxilla *in situ*, are so numerous that I desire to record the method which I have adopted in several cases with equally good results. The great advantages over other methods are the perfect rigidity of fractured portions, neatness, cleanliness, absence of a bulky splint and an earlier use of the maxilla for masticating purposes.

A powerful wherryman, aged nineteen, was admitted into the Leeds General Infirmary on May 11th, suffering from compound fracture of the lower jaw, etc. A gutta-percha splint and a four-tailed bandage were applied, but without effect; and on May 19th I was asked to see the case. I found the lower maxilla fractured transversely across the ramus on the patient's left side, also perpendicularly between the two fangs of the first molar on the same side, and likewise perpendicularly through the socket of the second bicuspid on the patient's right. The front portion was much displaced, being deeply depressed and exposing the second fang of the molar in its full length. The front portion was also considerably overlapping the posterior part, carrying the median line of the maxilla considerably over to the patient's left side. I took impressions in wax of both maxillas. On May 20th, the patient was anæsthetised, and with a dental engine and a specially made bayonet-shaped drill I made a hole through the jaw between the first and second molars on the patient's left side, holding an oral spoon on the inner side to guard the drill from piercing the root of the tongue. I next passed a stout silver wire through the drilled hole and bored a second hole between the fang of the canine and the first bicuspid, and having brought the wire through from the inside, twisted the ends together and tightened them with pliers, I bent the tail down flat, so as to be out of the way. The chief difficulty in these cases is to return the wire after having passed it through the first drill hole. The method I adopt is as follows: While drilling the hole I hold, or get a dresser to hold, an oral spoon on the inner side as a tongue guard. Having pushed the wire through into the bowl of the spoon, the latter curls it from the tongue and one can then seize it with a pair of forceps. I then bend a long loop on it, and having passed a fine wire through the second drill hole, attach it to the loop and draw it through. The oral spoon is a great help by safeguarding the tongue, turning the wire, and preventing the finger from being lacerated in feeling for the wire—an important factor in cases of a specific character. In addition to the suture I applied a Hammond's splint, which fixed the fractured portion on the patient's right, and finally a well-ventilated gutta-percha splint.

On May 26th, the parts were in a most satisfactory condition. There was no discharge and the patient was free from pain. On June 2d, the portions wired together were in perfect position, but the back teeth met before the front ones, probably owing to the fracture through the ramus. A broad cotton bandage (double thickness) was applied, the frontal portion being tightened in order to raise the body of the jaw. On the 11th, the teeth were well opposed to each other. The patient was free from pain, and all looked healthy. He was made an out-patient. On July 7th, the bandage and Hammond's splint and wire suture were removed. All was found firm and in good position. To avoid future trouble from the stumps on the patient's left side I removed them.

In a case which I attended some time ago, there was the same happy result. The inferior maxilla was divided in the median line to facilitate the removal of cancer from the floor of the mouth. There was difficulty in obtaining union in the ordinary way, but the application of a strong wire suture, applied as described, answered most efficiently, and there was rapid union.—*Lancet*, Dec. 3, 1892.

